# Metabolomics Changes in Meat and Subcutaneous Fat of Male Cattle Submitted to Fetal Programming

**DOI:** 10.3390/metabo14010009

**Published:** 2023-12-22

**Authors:** Arícia Christofaro Fernandes, Guilherme Henrique Gebim Polizel, Roberta Cavalcante Cracco, Fernando Augusto Correia Queiroz Cançado, Geovana Camila Baldin, Mirele Daiana Poleti, José Bento Sterman Ferraz, Miguel Henrique de Almeida Santana

**Affiliations:** 1 Department of Animal Science, College of Animal Science and Food Engineering, University of São Paulo (USP), Av. Duque de Caxias Norte, 225, Pirassununga 13635-900, SP, Brazil; guilherme.polizel@usp.br (G.H.G.P.); mhasantana@usp.br (M.H.d.A.S.); 2 Department of Veterinary Medicine, Faculty of Animal Science and Food Engineering, University of São Paulo (USP), Av. Duque de Caxias Norte, 225, Pirassununga 13635-900, SP, Brazil; mirelep@usp.br (M.D.P.); jbferraz@usp.br (J.B.S.F.)

**Keywords:** meat quality, metabolites, muscular and adipose development, pregnancy, prenatal supplementation

## Abstract

This study investigated changes in meat and subcutaneous fat metabolomes and possible metabolic pathways related to prenatal nutrition in beef cattle. For this purpose, 18 Nellore bulls were used for meat sampling and 15 for fat sampling. The nutritional treatments during the gestation were: NP—not programmed or control, without protein-energy supplementation; PP—partially programmed, with protein-energy supplementation (0.3% of body weight (BW)) only in the final third of pregnancy; and FP—full programming, with protein-energy supplementation (0.3% of BW) during the entire pregnancy. The meat and fat samples were collected individually 24 h after slaughter, and the metabolites were extracted using a combination of chemical reagents and mechanical processes and subsequently quantified using liquid chromatography or flow injection coupled to mass spectrometry. The data obtained were submitted to principal component analysis (PCA), analysis of variance (ANOVA), and functional enrichment analysis, with a significance level of 5%. The PCA showed an overlap between the treatments for both meat and fat. In meat, 25 metabolites were statistically different between treatments (*p* ≤ 0.05), belonging to four classes (glycerophospholipids, amino acids, sphingolipids, and biogenic amine). In fat, 10 significant metabolites (*p* ≤ 0.05) were obtained in two classes (phosphatidylcholine and lysophosphatidylcholine). The functional enrichment analysis showed alterations in the aminoacyl-tRNA pathway in meat (*p* = 0.030); however, there was no pathway enriched for fat. Fetal programming influenced the meat and fat metabolomes and the aminoacyl-tRNA metabolic pathway, which is an important candidate for the biological process linked to meat quality and related to fetal programming in beef cattle.

## 1. Introduction

Nutrition is one of the environmental factors that can most influence the phenotype, and that producers are able to handle with some ease. However, knowledge on the internal physiological factors that control the production, well-being, and quality of its products has not been fully elucidated [1]. There are several gaps in understanding the mechanisms for obtaining certain phenotypes in beef cattle. Meat production, for example, in its most literal and basic sense constitutes the formation and growth of the animal’s muscular and adipose tissue [2]. For this, the individual needs to have substrate for this formation, which comes from nutrition and tissue metabolism. These tissues originate during the animal’s pregnancy [3]. Therefore, the supply of nutrients from the cow during pregnancy is responsible for ensuring adequate nutritional support for the development of these tissues in the progeny [4].

Carcass traits and meat quality are influenced by several metabolic pathways, based on the sum of interactions between genotype, environment and the interaction of both [5]. The metabolome is defined as the complete set of small molecules (metabolites) that participate in metabolism [6]. Metabolites are the result of the complex interactions that occur between the genome and the environment, and the science that understands how this mechanism occurs is metabolomics [7]. Therefore, the advancement of molecular biology and the advent of metabolomics could contribute to animal research and to the understanding of complex biological systems, such as the interaction of metabolites and pathways resulting in the expression of phenotypic characteristics [8]. The application of metabolomics comes to identify and quantify the characteristic metabolome that serves as a substrate for the formation of tissues of interest, allowing gains in meat quality and quantity in herds [9]. 

Studies using metabolomics in meat have emerged in recent years [10,11], as well as studies of the metabolome between meat produced under different animal feeding conditions [12,13]. In the study by Zhang et al. [14], it is possible to find information on the relationship between metabolites and aspects of meat quality such as color, pH, tenderness, etc. The work of Antonelo et al. [15] uses metabolomics as a tool for identifying variations in meat tenderness. In the research by Zuo et al. [16], metabolomics is approached as a way to explain at the metabolic level how a characteristic of meat (water retention capacity) is different according to maturation times. And a review presented by Bischof et al. [17] compiles several other studies that evaluate how metabolomic changes occur in meat during the transformation of muscle into meat after slaughter for long periods. These are all recent works that show the importance of meat metabolomics.

Knowledge of the metabolomic profile in meat/fat helps to compose the observed phenotype, based on the distinction of metabolites, so the quantity and type of metabolite can determine the physiological characteristics of the muscle and the quality characteristics of the meat [18]. The observation of changes in progeny resulting from interventions during pregnancy is known as fetal programming; several studies have correlated fetal programming with the development of muscle and adipose tissues [3,19,20]. Some studies have already tested different feeding strategies and characterized the metabolome of animals destined for meat production [12,21,22,23,24,25]. However, few studies have investigated the consequences of prenatal nutrition on the metabolism of their offspring [26,27,28,29].

This study aims to evaluate the meat and subcutaneous fat metabolome of young bulls submitted to fetal programming. Our hypothesis was that protein-energy supplementation at different periods of gestation or its absence in bovine cows alters the muscle and adipose metabolome of male progeny.

## 2. Methodology

### 2.1. Declaration of Ethics for the Use of Animals in Experimentation

All experimentation protocols involving these animals were previously approved by the Research Ethics Committee of the Faculty of Animal Science and Food Engineering of the University of São Paulo (under protocol CEUA n° 1843241117, on 10 March 2018), in accordance with the guidelines of the National Council for the Control of Animal Experimentation (CONCEA). 

### 2.2. Experimental Design 

A sample of 126 Nellore females was submitted to fixed-time artificial insemination using semen from four bulls. The females were multiparous and were 3–8 years old. After confirmation of pregnancy, the cows were selected and randomly assigned to treatments according to age, body weight, and body condition score, measured at the time of insemination in order to keep the batches as homogeneous as possible. All cows remained in paddocks with pasture of *Brachiaria brizantha* cv. Marandu throughout the experiment. All females received mineral supplementation (0.03% average body weight of each batch) and water ad libitum throughout pregnancy.

Each cow was considered an experimental unit and was assigned to a completely randomized design with three treatments, being prenatal nutrition strategies (i.e., fetal programming), which consisted of providing a protein-energy supplementation during pregnancy. The treatments were: not programmed or control (NP), without protein-energy supplementation; partially programmed (PP), with supplementation only in the final third of pregnancy; and full programming (FP), with protein-energy supplementation throughout the pregnancy until calving. Both the PP and FP groups received a daily supplement corresponding to 0.3% of the average body weight of the cows until calving, in accordance with the National Research Council’s (NRC, 2000) [30] nutritional, maintenance, and gain recommendations for cows during mid- to late pregnancy. The ingredients and nutrients in the supplement offered to pregnant cows and the nutrients in pastures can be found in the methodology of Schalch Junior et al. [26]. 

After calving, all animals received the same sanitary, nutritional, management, and experimental collection conditions. All male progeny (63 animals, 21 per treatment) spent 8 months with the mother until weaning and another 11 months in the rearing phase on pasture and supplementation in the trough, followed by an average of 106 days of a finishing phase in the feedlot. At calving, all animals received conventional care (weighing, navel healing, deworming, and identification tattoo) and were kept under the same pasture system (rotated in *Brachiaria brizantha* cv. Marandu) staying with their dams until weaning. Afterward, they passed to the rearing phase, under the same forage species but in two paddocks, and additionally now receiving an energy supplementation of 0.3% of the average weight of the lot during the dry period and protein supplementation of 0.1% of the average weight of the lot in water during the rearing period until the animals entered the feedlot.

The animals were divided into two collective pens according to the entry weight upon arriving at the feedlot facilities and were slaughtered after the finishing period. The slaughter was carried out in accordance with humanitarian procedures, as required by Brazilian law [31,32]. The slaughter was carried out in compliance with humane procedures, as required by Brazilian law. The slaughter procedure occurred in three groups of 21 animals each (7 animals per treatment) and with the slaughter of one group per week, but all 21 animals of each group were slaughtered on the same day. The selection criterion of the animals for each slaughter group was the subcutaneous fat thickness (SFT) obtained in the last carcass ultrasound on the penultimate day of the finishing phase (pre-slaughter). Based on these data, slaughter began with the group of animals with the highest SFT in each treatment. After slaughter, the carcasses were placed in refrigeration chambers with a temperature between −4 and 0 °C for 24 h.

The males were subjected to evaluations involving body weight measurements from birth to slaughter. Only during weaning (8 months) were the groups differentiated. During this period, the PP and FP groups demonstrated a greater body weight compared to the NP group (control), although this distinction was not manifested at any other time, as reported by Cracco et al. [33]. The measurements of the groups’ weights over the period can also be consulted in the cited work.

The animals used for tissue metabolomics were selected from a subsample, more specifically 18 males for meat metabolomics, 6 of each treatment, while 15 of the 18 animals (5 from each treatment) were selected for fat metabolomics. To ensure greater data homogeneity, the animals were selected based on the premise that they were offspring of the same father and that the mothers were born in the same year. 

### 2.3. Tissue Collection

Samples of meat and adipose tissue were used to carry out the analyses. The meat tissue samples were obtained from the *Longissimus thoracis et lumborum* muscle, on the sectioned face between the 12th and 13th rib, and the subcutaneous fat from this same muscle and in the same position; both were obtained 24 h after slaughter.

In the deboning room, the sirloin steak was separated from the carcass and arranged for the seizure of biological material. Both tissues were collected at the time of deboning and handled as hygienically and quickly as possible. The samples were aliquoted and placed individually in liquid nitrogen in previously autoclaved aluminum foil packaging. The meat samples were removed in a similar position between the animals, at the central point of the cut and the fat layer, on the sectioned face between the 12th and 13th rib. Small samples were removed with tweezers and a scalpel, which were sanitized and free of cross-contamination, and then stored in an ultrafreezer at −80 °C until extraction. 

### 2.4. Tissue Homogenization and Metabolite Extraction 

According to the methodology proposed by Zukunft et al. [34], for skeletal muscle and fat tissues, there is a specific solvent and an indicated proportion. In the case of the solvent (same for both tissue), each type of tissue was homogenized in two different reagents: 10 mM phosphate buffer pH 7.5 at 25 °C and ethanol 85/15 (*v*/*v*)/10 mM mixture of phosphate buffer pH 7.5 (EtOH/PB). As for the proportions [mg of tissue to X µL of solvent, denoted by 1:X (*w*/*v*)], the proportion 1:3 was used for muscle and 1:6 for fat.

### 2.5. Preparation of Extraction Solvent 

A mixture of the two reagents mentioned above and water was used to compose the total extraction solvent. Initially, we used solvent C composed of 10 mL of 0.1 M phosphate buffer +90 mL of water (HPLC). For the formation of solvent A, 15 mL of solvent C was added to 85 mL of ethanol (HPLC-grade), forming the specific solvent for the extraction of muscle and fat samples. 

The samples were removed from the aluminum foil and weighed for homogenization, and 30 g was the tissue weight used for extraction. Then, the samples were placed in pre-cooled cryotubes (dry ice) containing ceramic spheres with a diameter of 1.4 mm (Precellys Homogenization Kit, CK14, PEQLAB Biotechnology, Erlangen, Germany). Subsequently, the appropriate solvent proportion of each tissue was added into the cryovials, with 30 g of meat/90 mL of solvent and 30 g of fat/180 mL of solvent.

After freezing at −20 °C, the extraction solvent was added to the cryotubes with the samples that were previously weighed. Then, the samples were homogenized in a Precellys^®^24 homogenizer equipped with an integrated cooling unit (PEQLAB Biotechnology, Erlangen, Germany) three times for 20 s at 5500 rpm with 30 s intervals to ensure constant temperatures during homogenization [34].

After, the samples were centrifuged at 10,000 rpm for 5 min at +4 °C to separate the metabolites. The supernatant generated from these procedures was pipetted into a new vial and stored at −80 °C. For the metabolomic analysis, 10 µL of the supernatant was used. 

### 2.6. Targeted Metabolomics

The metabolomic analysis was carried out by the company Apex Science (Campinas, São Paulo, Brazil). The AbsoluteIDQ ^®^Kit p180 (Biocrates Life Sciences AG, Innsbruck, Austria) was the product used for the metabolite quantification. The kit comprised 188 metabolites, 21 of which were amino acids, 21 biogenic amines, 40 acylcarnitines (Cx:y), 14 lysophosphatidylcholines (lysoPC), 76 phosphatidylcholines (PC), and 15 sphingolipids (SMx:y), where “x” represents the number of carbons and “y” the double bonds of all chains. The amino acids and biogenic amines were derivatized using phenylisothiocyanate. These classes of metabolites were analyzed using liquid chromatography–tandem mass spectrometry (HPLC–MS/MS) using an AB Sciex 4000 QTRAP mass spectrometer (AB Sciex, Darmstadt, Germany) with electrospray ionization. The lysophosphatidylcholines, phosphatidylcholines, acylcarnitines, and hexose were analyzed using injection flow–tandem mass spectrometry (FIA–MS/MS).

To carry out the data analysis of metabolite quantification and quality assessment, the MetIDQ^®^ v1.0 software (part of the AbsoluteIDQ^®^ p180 kit) was used. The metabolite concentrations (measured in µM) were calculated using the internal standards. The enterprise Biocrates Life Sciences AG determined experimentally the metabolite-specific detection limits (LOD) of the assay. 

The metabolite quantification methodology, kit validation, internal standards for the liquid chromatography method, and concentrations of reference analytes for quality control (QC1–3), carried out by Apex Science, were replicated from Zukunft et al. [34], being a protocol established by the commercial kit supplier and adapted for tissues. 

### 2.7. Statistical Analysis

The data processing and univariate analysis (analysis of variance—ANOVA) of the metabolites were performed in the R software environment (version 4.1.2). Metabolites with more than 70% of samples below the LOD or with the same values across samples were removed from the dataset. The LOD values that remained in the metabolome after filtering were replaced by the mean of each variable. The model was implemented using the “LM” function in R.

The statistical model used for the metabolomic and phenotypic analyses of bulls was:

Yjk = μ + β1Ageb1 + Tratj + εjk

where Yjk is the observed metabolite of the kth animal, recorded in the jth treatment; μ is a constant; β1 is the regression coefficient of the animal age covariate; Ageb1 is the observed value for the animal age of the kth animal; Tratj is the fixed effect of the jth treatment; and εjk is the residual random term. The residuals were tested for normality (Shapiro–Wilk test) and homoscedasticity (Levene test) and differences between treatments were considered significant when *p* ≤ 0.05 according to the Tukey–Kramer test.

In addition, the concentration of metabolites was analyzed using the MetaboAnalyst 5.0 software and the data were auto-sized (centered on the mean and divided by the standard deviation of each variable) before analysis. We performed the principal component analysis (PCA) and the functional enrichment analysis of metabolic pathways for the significant metabolites in the meat and fat samples. The PCA was performed to evaluate the clustering between treatments (NP, PP, and FP). The enrichment analysis was performed to identify the most relevant biological processes associated with the differentially expressed metabolites (identified in the univariate analysis) based on the Kyoto Encyclopedia of Genes and Genomes database (KEGG Pathway) and PubChem (Open Chemical Database on National Institutes of Health (NIH)). These databases allow you to find different biological pathways (e.g., energy, lipid, and amino acid metabolism) related to input (differential expressed metabolites) and molecule functionality. Biological processes with a *p* value ≤ 0.05 were considered significant.

## 3. Results

### 3.1. Principal Component Analysis (PCA)

**Figure 1:**
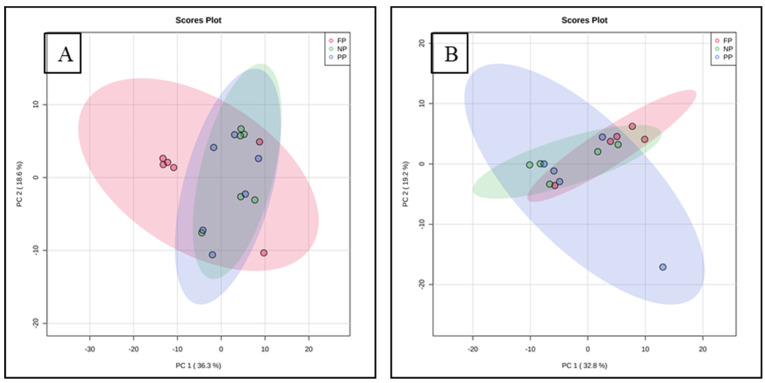
Principal component analysis (PCA) of meat (**A**) and subcutaneous fat; (**B**) metabolome of males programmed during gestation.

The distribution of all data analyzed showed an overlap between all groups and there was no clustering between the treatments. The overlap indicates similarity between the treatments. The two principal components together for meat explain 54.9% of the total variance (PC1 = 36.3%; PC2 = 18.6%), whereas for fat, 52.0% of the total variance is explained (PC1 = 32.8%; PC2 = 19.2%) (Figure 1).

### 3.2. Heatmaps 

Heatmaps allow you to visualize the global metabolomic profile of meat and fat and its variations between groups. And they represent a visual summary of the metabolites affected by treatments. The differentially expressed metabolites between the groups can be seen from Figure S1 (meat) and Figure S2 (fat) below.

### 3.3. Meat Metabolites 

From the set of 188 metabolites evaluated, 25 metabolites were differentially expressed between treatments in meat (*p* ≤ 0.05; 18 metabolites belonging to the class of phosphatidylcholines, 3 of amino acids, 2 of sphingolipids, 1 of biogenic amine, and 1 of lysophosphatidylcholine; (Table 1). 

Regarding the class of phosphatidylcholines, the metabolites PC aa C26:0; PC aa C34:2; PC aa C38:0; and PC ae C36:2 had lower concentrations in the FP treatment compared to the NP and PP treatments (*p* ≤ 0.05). Conversely, in the PP treatment, there was a higher concentration compared to the FP treatment for the following metabolites: PC ae C34:2; PC ae C40:1; PC ae C38:5; PC ae C36:5; PC ae C36:1; PC ae C40:6; PC aa C32:2; PC aa C42:0; PC ae C44:6; and PC ae C36:4 (*p* ≤ 0.05). The metabolites PC ae C34:1; PC aa C34:3; PC ae C32:1; and PC aa C36:2 showed a lower concentration in the FP treatment compared to the NP treatment (*p* ≤ 0.05).

In the amino acid class, the threonine (*p* = 0.032) and arginine (*p* = 0.044) levels were higher in treatments PP and FP, but alanine (*p* = 0.022) was higher in the PP group in relation to the NP group. In the class of sphingolipids, SM C18:0 (*p* = 0.018) was lower in FP compared to PP and SM (OH) C24:1 (*p* = 0.024) lower in FP compared to NP. For the metabolite lysoPC at C26:1 (*p* = 0.031), belonging to the lysophosphatidylcholine class, a smaller amount was found in FP, relative to NP and PP in meat. In the class of biogenic amines, spermidine (*p* = 0.022) had a higher expression in the treated groups (PP and FP).

### 3.4. Subcutaneous Fat Metabolites 

From the set of 188 metabolites evaluated in this study, 10 metabolites were differentially expressed between treatments in the subcutaneous fat (*p* ≤ 0.05). These different metabolites comprise nine in the phosphatidylcholine class and one in lysophosphatidylcholine (Table 2).

Regarding the metabolites PC ae C40:5; PC aa C38:3; PC aa C38:5; and PC aa C40:6 of the phosphatidylcholine class, the values obtained from the FP treatment were higher than from NP (*p* ≤ 0.05). As for PC aa C36:0; PC ae C36:0; and PC ae C38:0, the FP treatment showed higher values than the PP treatment (*p* ≤ 0.05). A significant difference for PC aa C36:1 and PC ae C30:2 was found in the higher expression with FP compared to with NP and PP (*p* ≤ 0.05).

In the lysophosphatidylcholine class, lysoPC at C26:1 (*p* = 0.029) had its expression increased in PP compared to the control group (NP), but did not differ from FP.

### 3.5. Enrichment Analysis

**Figure 2:**
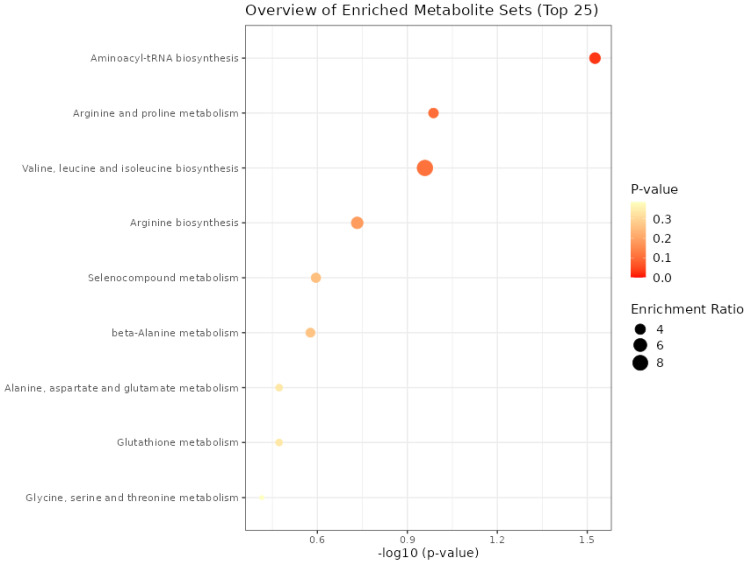
Metabolic processes expressed in the meat of males submitted to fetal programming (NP, PP and FP).

The aminoacyl–transporter RNA biosynthesis pathway was significant (*p* = 0.03) only for the metabolites present in meat from the functional enrichment analysis of metabolic concentration data using the Kyoto Encyclopedia of Genes and Genomes (KEGG) pathway (Figure 2). For the other processes, it was not possible to find a difference between the groups; even though the ratio of enrichment of the valine, leucine, and isoleucine biosynthesis pathway proved to be greater than the aminoacyl–tRNA biosynthesis, it was not enough to present a significant *p* value.

## 4. Discussion

There were many altered metabolites in the tissues and the classes to which they belonged were selected for discussion.

Amino acids are the small units that comprise proteins and protein is the most valuable meat component from a nutritional and marketing viewpoint [35]. Threonine, arginine, and leucine are examples of the amino acids in beef [36]. 

In our study, we found significance in the threonine and arginine levels, as we obtained a greater expression of these metabolites in treatments with prenatal nutritional stimulation, indicating that supplementation during pregnancy alters the profile of Thr and Arg in the meat of male progeny. As for the third amino acid found, alanine (Ala), we observed a difference only in the group that received supplementation in the final third of pregnancy (PP treatment).

Corroborating our results, Kwon et al. (2004) [37] found that food restriction throughout pregnancy causes a decrease in the same three amino acids mentioned above (Thr, Arg, and Ala). This shows that nutrient prenatal supplementation during gestation impacts the amino acid metabolism of the offspring at slaughter. Similarly, Hellmuth et al. (2016) [38] found effects of maternal nutritional restriction on the energy metabolism in baboons, with increased hepatic glucose concentration and decreased plasma levels of the amino acids methionine and threonine and hepatic threonine in the offspring that underwent restriction.

The class of sphingolipids is the second largest in terms of membrane lipids in animals and plants [39]. The metabolites SM C18:0 and SM(OH) C24:1 are part of a subcategory of sphingolipids called sphingomyelin. Sphingomyelins participate in cell proliferation, extracellular and intracellular signaling, cell differentiation, autophagy, and apoptosis [40]. From a nutritional viewpoint, the presence of sphingolipids in meat inhibits colon carcinogenesis, decreases serum LDL cholesterol, and raises HDL, suggesting that these metabolites represent a functional food constituent [41,42]. Our results show that prenatal supplementation throughout the entire gestation (FP treatment) increased this class of metabolites in relation to the control group or the PP treatment. Therefore, constant stimulus with nutrients during pregnancy ensures the benefits of this compound for the animal and for the consumer. 

Phosphatidylcholines and lysophosphatidylcholines are part of the class of Glycerophospholipids, which is the main constituent of the mitochondrial membrane. This means that the more these metabolites are found in meat or fat, the more mitochondria are likely to be found [43]. Consequently, there may be a greater oxidation of these tissues since the function of mitochondria is to promote oxidation to obtain energy. The quality of a meat product with a large number of glycerophospholipids and that remains exposed on meat shelves at retail stores for a longer time may have its quality compromised by the oxidation process of lipids and by a rancid flavor [44]. In our study, the meat of the animals from the control group contained a larger number of glycerophospholipids. In the subcutaneous fat, however, we see the opposite, with a greater presence of glycerophospholipids in the fat of animals with prenatal nutritional stimulation.

Similarly, Muroya et al. (2021) [29] found changes in the glycerophospholipid expression pathway in the muscle of bovine fetuses from malnutrition during pregnancy. These modifications may interfere with postnatal muscle growth and repair [45]. 

Biogenic amines, such as spermidine, are metabolites formed by nitrogenous bases from the decarboxylation of free amino acids by microbial action, and are considered antinutritional [46]. This classification has two important motivations in terms of meat consumers since the food can have different levels of biogenic amines [39]. First, the health risk to humans due to the toxicity of biogenic amines and possible drug interactions is an issue [47,48]. Second, the quality and acceptability of the food are compromised [49]. As the level of amines depends on the action of bacteria, which can be toxic, the metabolite can be observed to indicate food freshness. The greater the production of biogenic amines, the larger the number of microorganisms and the more deteriorated the food becomes [50]. Furthermore, spermidine, which is categorized as a polyamine, can be altered by physiological stimuli or by inhibitors of metabolic enzymes [51]. Our work indicated that prenatal nutritional stimulus of cows increased the meat spermidine concentration of male offspring, which may indicate a possible worsening of meat quality. In pregnant cows that underwent nutritional restriction, an increase in the spermidine precursor was also observed, possibly altering the metabolism of this polyamine in the offspring [29].

Functional enrichment analysis aims to identify changes in the expression of pathways that comprise the biological processes, both when increased and decreased or when associated with the physiological responses of organisms. In the present study, nine processes were found; however, only one was significant among the treatments, with increased expression in the aminoacyl–transporter RNA biosynthesis pathway. 

Biosynthesis is a chemical process that involves the production of various molecules in a living organism, such as lipids, nucleic acids, and proteins, from simple compounds. Simple reactants cluster to convert another compound or to build macromolecules [52]. The union/conversion process of these molecules comes from metabolic pathways. Metabolic pathways are responsible for receiving and transmitting substrates to other reactions; therefore, they are dependent on other pathways to occur. The aminoacyl–tRNA molecule is a transport enzyme that, along with the translation factors, carries amino acids to the ribosomes that incorporate these amino acids into peptide chains for protein formation [53]. Thus, the increase in the pathway responsible for the biosynthesis of aminoacyl–tRNA translates into more reactions occurring in the meat of the animals in the PP and FP treatments for the formation of aminoacyl–tRNA enzymes, which is positive since the latter have the function of providing varied proteins to the cell membrane, such as the proteins that make up muscle. The process becomes even more advantageous when the activation of metabolic pathways, such as this one, happens during pregnancy, as the moment of formation and growth of new tissues happens in that period and can modulate the results throughout the animal’s life [54]. 

The discoveries conferred by the use of new technologies in the present study offer a better understanding of physiological processes and provide excellent advances in beef production as a whole [14]. The continuous development of new technologies and methods for complex data analyses using modeling and prediction of functional biochemical networks allows us to approach the general biology that combines molecular, genetic, and environmental data, contributing to better understanding the phenotype of interest [55].

## 5. Conclusions

The prenatal nutrition of Nellore cows was able to modify the levels of some metabolites present in the meat and subcutaneous adipose tissue of the male offspring. Furthermore, a significant metabolic process was found in the meat metabolome to be correlated with different supplementation strategies during pregnancy (the aminoacyl–transporter RNA biosynthesis pathway). Thus, maternal nutrition can modulate pathways and metabolites that culminate in phenotypic changes in meat and fat, which can alter the animal’s performance characteristics and/or the quality of the final product for the consumer.

## Figures and Tables

**Table 1:** Significant metabolites in meat from male cattle supplemented during pregnancy.

Metabolites	NP	PP	FP	*p* Value ^1^
PC aa C26:0	0.65 ± 0.14 ^a^	0.78 ± 0.13 ^a^	0.36 ± 0.21 ^b^	0.003
PC ae C34:2	3.17 ± 1.39 ^ab^	5.91 ± 2.89 ^a^	1.35 ± 1.71 ^b^	0.010
PC ae C40:1	0.19 ± 0.13 ^ab^	0.41 ± 0.18 ^a^	0.14 ± 0.18 ^b^	0.012
PC ae C34:1	6.33 ± 2.26 ^a^	4.70 ± 2.01 ^ab^	1.62 ± 2.06 ^b^	0.013
PC aa C34:2	100.38 ± 60.14 ^a^	94.48 ± 33.05 ^a^	18.24 ± 26.06 ^b^	0.017
PC ae C38:5	3.77 ± 1.87 ^ab^	5.03 ± 2.51 ^a^	1.33 ± 1.82 ^b^	0.017
SM C18:0	10.25 ± 4.15 ^ab^	14.15 ± 3.40 ^a^	4.55 ± 5.69 ^b^	0.019
Espermidine	0.35 ± 0.09 ^b^	0.49 ± 0.13 ^a^	0.52 ± 0.06 ^a^	0.022
Ala	864.56 ± 118.87 ^b^	1061.35 ± 74.07 ^a^	981.71 ± 147.33 ^ab^	0.022
SM (OH) C24:1	0.59 ± 0.17 ^a^	0.41 ± 0.16 ^ab^	0.22 ± 0.24 ^b^	0.024
PC ae C36:5	3.77 ± 1.55 ^ab^	5.35 ± 2.66 ^a^	1.58 ± 2.09 ^b^	0.025
PC aa C38:0	1.35 ± 0.55 ^a^	1.28 ± 0.38 ^a^	0.45 ± 0.66 ^b^	0.026
PC ae C36:1	9.42 ± 3.51 ^ab^	11.49 ± 6.88 ^a^	2.82 ± 4.13 ^b^	0.027
lisoPC a C26:1	0.29 ± 0.13 ^a^	0.30 ± 0.10 ^a^	0.10 ± 0.15 ^b^	0.031
Thr	61.53 ± 9.96 ^b^	80.00 ± 10.63 ^a^	78.21 ± 11.78 ^a^	0.032
PC ae C40:6	1.23 ± 0.56 ^ab^	1.78 ± 0.79 ^a^	0.60 ± 0.88 ^b^	0.034
PC aa C32:2	7.42 ± 3.39 ^ab^	10.42 ± 7.10 ^a^	1.91 ± 3.52 ^b^	0.035
PC aa C42:0	0.06 ± 0.03 ^ab^	0.09 ± 0.04 ^a^	0.04 ± 0.03 ^b^	0.038
PC aa C34:3	8.94 ± 4.69 ^a^	7.60 ± 3.76 ^ab^	2.12 ± 2.97 ^b^	0.040
PC ae C32:1	1.89 ± 0.95 ^a^	1.60 ± 0.80 ^ab^	0.48 ± 0.59 ^b^	0.040
PC ae C36:2	19.28 ± 11.68 ^a^	19.43 ± 5.84 ^a^	5.16 ± 8.17 ^b^	0.041
PC aa C36:2	288.65 ± 162.68 ^a^	180.22 ± 118.81 ^ab^	64.00 ± 94.01 ^b^	0.043
Arg	45.40 ± 11.59 ^b^	59.03 ± 8.93 ^a^	59.62 ± 9.35 ^a^	0.044
PC ae C44:6	0.11 ± 0.04 ^ab^	0.16 ± 0.05 ^a^	0.09 ± 0.04 ^b^	0.045
PC ae C36:4	4.66 ± 3.02 ^ab^	7.54 ± 4.99 ^a^	1.29 ± 2.01 ^b^	0.049

^1^—*p* value between treatments. The small letters superscripted represent the significant contrasts. NP—not programmed; PP—partially programmed; FP—full programming.

**Table 2:** Significant metabolites of subcutaneous fat from males submitted to fetal programming.

Metabolites	NP	PP	FP	*p* Value ^1^
PC aa C36:0	2.06 ± 2.89 ^ab^	1.48 ± 1.01 ^b^	7.39 ± 5.11 ^a^	0.013
PC ae C40:5	0.87 ± 0.84 ^b^	1.19 ± 0.77 ^ab^	2.32 ± 1.09 ^a^	0.016
PC ae C36:0	1.64 ± 2.52 ^ab^	0.77 ± 0.49 ^b^	4.43 ± 2.88 ^a^	0.019
PC ae C38:0	1.32 ± 1.51 ^ab^	1.08 ± 0.64 ^b^	3.24 ± 1.55 ^a^	0.027
PC aa C38:3	7.23 ± 9.04 ^b^	7.83 ± 8.98 ^ab^	23.52 ± 14.35 ^a^	0.031
PC aa C38:5	7.12 ± 8.26 ^b^	9.12 ± 8.92 ^ab^	19.84 ± 12.54 ^a^	0.032
PC aa C36:1	43.79 ± 43.77 ^b^	35.70 ± 33.50 ^b^	143.48 ± 83.53 ^a^	0.034
PC ae C30:2	0.40 ± 0.35 ^b^	0.41 ± 0.35 ^b^	1.11 ± 0.49 ^a^	0.042
PC aa C40:6	2.65 ± 3.74 ^b^	2.92 ± 3.77 ^ab^	9.15 ± 8.32 ^a^	0.048
lysoPC a C26:1	0.11 ± 0.10 ^b^	0.25 ± 0.15 ^a^	0.21 ± 0.07 ^ab^	0.049

^1^—*p* value between treatments. The small letters superscripted represent the significant contrasts. NP—not programmed; PP—partially programmed; FP—full programming.

## Data Availability

None of the data were deposited into an official repository. The raw data supporting the conclusion of this article will be made available by the authors without undue reservation.

## Supplementary Materials

The following supporting information can be downloaded at https://www.mdpi.com/article/10.3390/metabo14010009/s1, Figure S1: Metabolomic profile of meat from bulls supplemented during pregnancy; Figure S2: Metabolomic profile of subcutaneous fat in bulls that received prenatal nutrition.
