# Supplementary figures and images for: Metabolomics Changes in Meat and Subcutaneous Fat of Male Cattle Submitted to Fetal Programming
Source: Metabolites. 2023 Dec 22;14(1):9. doi: 10.3390/metabo14010009 (PMC10819762; doi:10.3390/metabo14010009)

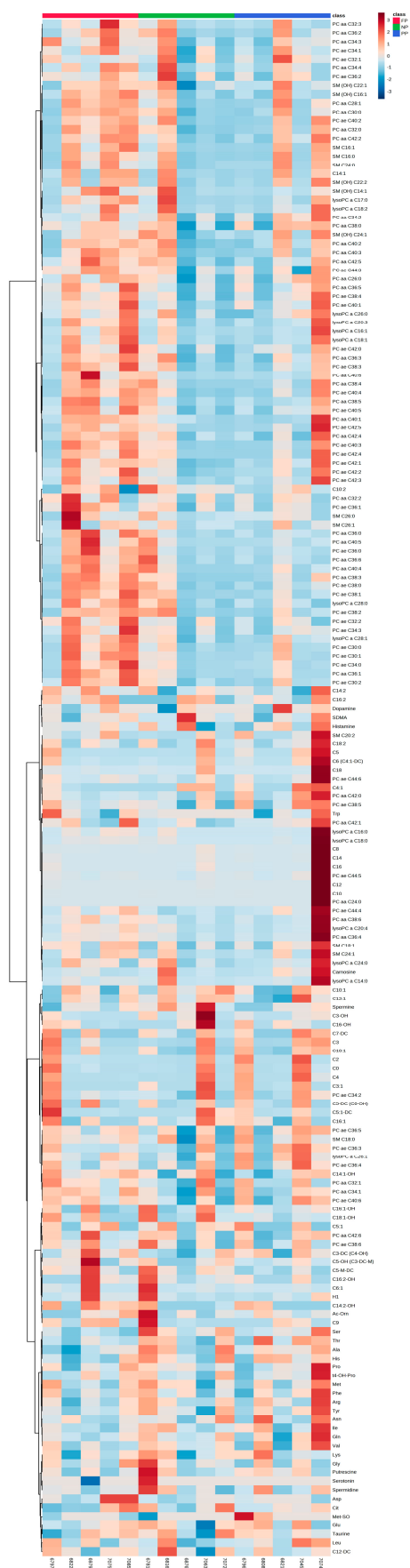

Figure S2. Metabolomic profile of subcutaneous fat in bulls that received prenatal nutrition.

Supplement: Supplementary file 1 [file metabolites-14-00009-s001.zip › metabolites-2771464-supplementary.pdf]
